# Exploring the Structure of Human Defensive Responses from Judgments of Threat Scenarios

**DOI:** 10.1371/journal.pone.0133682

**Published:** 2015-08-21

**Authors:** Laura A. Harrison, Curie Ahn, Ralph Adolphs

**Affiliations:** 1 Computation & Neural Systems, California Institute of Technology, Pasadena, California, United States of America; 2 Biology, California Institute of Technology, Pasadena, California, United States of America; 3 Humanities & Social Sciences, California Institute of Technology, Pasadena, California, United States of America; Birkbeck, University of London, UNITED KINGDOM

## Abstract

How humans react to threats is a topic of broad theoretical importance, and also relevant for understanding anxiety disorders. Many animal threat reactions exhibit a common structure, a finding supported by human evaluations of written threat scenarios that parallel patterns of rodent defensive behavior to actual threats. Yet the factors that underlie these shared behavioral patterns remain unclear. Dimensional accounts rooted in Darwin’s conception of antithesis explain many defensive behaviors. Across species, it is also clear that defensive reactions depend on specific situational factors, a feature long emphasized by psychological appraisal theories. Our study sought to extend prior investigations of human judgments of threat to a broader set of threats, including natural disasters, threats from animals, and psychological (as opposed to physical) threats. Our goal was to test whether dimensional and specific patterns of threat evaluation replicate across different threat classes. 85 healthy adult subjects selected descriptions of defensive behaviors that indicated how they would react to 29 threatening scenarios. Scenarios differed with respect to ten factors, e.g., perceived dangerousness or escapability. Across scenarios, we correlated these factor ratings with the pattern of defensive behaviors subjects endorsed. A decision tree hierarchically organized these correlation patterns to successfully predict each scenario’s most common reaction, both for the original sample of subjects and a separate replication group (n = 22). At the top of the decision tree, degree of dangerousness interacted with threat type (physical or psychological) to predict dimensional approach/avoidance behavior. Subordinate nodes represented specific defensive responses evoked by particular contexts. Our ecological approach emphasizes the interplay of situational factors in evoking a broad range of threat reactions. Future studies could test predictions made by our results to help understand pathological threat processing, such as seen in anxiety disorders, and could begin to test underlying neural mechanisms.

## Introduction

Darwin famously noted the striking phylogenetic continuity of emotional behaviors, including responses to threat [1]. Defensive behaviors, ranging from flight to attack, have evolved to deal with environmental challenges that show a common structure across all animals: the need to attack an aggressor, to flee a predator, or to hide from an inescapable threat, to name only a few prototypical situations. Over the years, several empirical and theoretical studies, largely rooted in biology and ethology, have supported the idea of common structure in defensive behaviors across species, ranging from rodents to humans [2]. Various schemes have been proposed for how these are organized, ranging from ethologically-identified [3] factors like risk assessment [4] to dimensional accounts including threat imminence [5] and a classic approach/avoidance account whereby all motivated/emotional behaviors are organized along an appetitive and defensive system [6].

On the other hand, the literature in affective psychology has rarely incorporated specific details of the data from nonhuman animals, although this literature clearly does acknowledge the biological roots of human defensive behaviors [7–10]. Here, we asked people to select hypothetical defensive behaviors to descriptions of a range of physically threatening situations, as well as to situations of social psychological threat. It is important to emphasize at the outset that we rely on verbal report and ratings, as is common in many psychological studies in humans (e.g., [11]), rather than on actual observed defensive behavior. Verbal report to hypothetical scenarios by humans has been found in previous studies to correlate with actual rodent behavior patterns across three laboratories [4], and we used it here as a first approach to assess responses for which live exposure would be ethically difficult to obtain. Specifically, in the current experiment, threatening situations include situations of social psychological threat, (e.g., blackmail), social physical threat (e.g., stalking), as well as physical threat from other species and natural disasters. Inclusion of these different threat categories underlies an attempt to bridge our understanding of basic approach-avoidance reactions to predators and other physical threats, on the one hand, with a characterization of defensive reactions to less physical but more psychological intra-species threats that relate to issues of social inclusion, social hierarchies, and social dominance, on the other hand. It is worth noting that socially modulated threat reactions have been observed across diverse phylogenetic classes, including fish [12], and mammals [13] ranging from rodents [14] to primates [15, 16].

Defensive behaviors in rodents and primates have been extensively studied, and related to human behavior, such as in the case of humans physically freezing in response to threatening stimuli [17]. Innate patterns of defensive behavior have been identified in some detail in rats: e.g. high magnitude threats elicit a flight response, only if an escape route is available; if an escape route is not available, rodents will freeze, show a defensive threat (e.g., vocalization), or launch an explosive defensive attack depending on the distance of the threat [18]. Very specific releasing-stimulus like cues can be sufficient to trigger the behavior: for instance, a predator-like visual looming stimulus (just an expanding black circle on the ceiling) is sufficient to produce robust freeze or flight [19], with the likelihood of each behavior dependent upon the presence of a hiding place in the arena. The size of an enclosure also seems to affect the use of flight or freeze behavior [20]. The validity of the use of rodent defensive behaviors as a model for human defensive reactions remains an open question, partially addressed by a study that attempted to make direct comparisons between the two species [2]. In that study, written descriptions of physically threatening scenarios were manipulated in terms of factors known to alter rodent behavior, such as the magnitude of threat, escapability of the situation, ambiguity of the threat stimulus, distance between the threat and the subject, and the presence of a hiding place. Strikingly, most of the human subjects’ choices of what they would do when faced with these scenarios paralleled the rodent behavior observed when a rat faced the same real situational factors. Moreover, human choices of defensive behaviors paralleled patterns of animal defensive behaviors (e.g., defensive attack for near threats; risk assessment for ambiguous threats; hiding when there is a hiding place) across different cultural settings (cf. Table 6, Discussion). Patterns similar to those originally observed in Hawaiian subjects [2] were observed in Brazil [21] and Wales [22] with only a few “minor or potentially easily explained differences” [4], suggesting cross-cultural generality at least for the physically threatening scenarios investigated in those studies.

These prior studies that built upon rodent behaviors fit well with dimensional accounts of emotion. Although Darwin is often cited in support of discrete emotion theories, Darwin’s early principle of antithesis [1] in fact set the framework for conceiving of emotional behaviors as having a dimensional structure:

When actions of one kind have become firmly associated with any sensation or emotion, it appears natural that actions of a directly opposite kind…should be unconsciously performed…under the influence of a directly opposite sensation or emotion. (p. 67)

Darwin’s notion of antithesis roughly maps onto the modern dimension of “valence”. However, the main point that he made, of course, was that emotions, including defensive behaviors, in humans would look similar and have a similar structure to those of other mammals. According to one theory, evolutionary selection can give rise to what have been called “rules of thumb” that advantageously guide behavior under typical ecological conditions [9]. These rules of thumb can be conserved across species that have evolved in similar environments, such that emotional behaviors evoked by certain circumstances in one species will evoke similar emotional behaviors in another species faced with the same challenges. If the species are not too phylogenetically distant, one would even expect these shared emotional responses to be mirrored in conserved neural structures [23, 24]. It is unknown precisely which features of a shared environment would come into play in this picture, but there are some good candidate dimensions, such as predator imminence (the physical distance and time to discovery between predator and prey) [5] and uncertainty. Notably, these dimensions are broad, can be observed across many species and provide important context for many situations.

However, in addition to such broad dimensional structure, it is clear that emotions also exhibit patterns of response tailored to specific situations that evoke them. For instance, Gray and McNaughton [25] have proposed that two clusters of defensive behaviors identified also in rodent studies [26, 27] represent the action of two brain systems, one controlling anxiety, the other fears, and that differences in the reactivity of these systems give rise to personality differences and ultimately could explain psychopathology [8, 25]. It has been proposed that different circuits involving the amygdala and the bed nucleus of the stria terminalis (BNST) mediate phasic fear versus more sustained anxiety-like fear [28]. The distinction between anxiety and fear is important, mapping onto those defensive situations where engagement and the acquisition of further information is adaptive (in the former case), and those where disengagement and survival are most important (in the latter case).

Social fear is yet another category, linked to a possibly domain-specific class of eliciting stimuli. There is evidence that social fear is processed differently from other types of fear: in mice, independent hypothalamic circuits for social (intra-species) and predator (inter-species) fear have been identified [29]. Do patterns of threat response observed in other species extend to the social domain in humans, especially to more psychological as opposed to physical social threat scenarios? Although the Blanchard study [2] and its replications [21, 22] investigated physical threat between humans, psychological threat has rarely been directly compared to physical social threat. Social sources of threat have been studied experimentally in humans with paradigms such as the Trier Social Stress Test [30], or the cyberball game [31], which relates to ostracism and social hierarchy, issues that have been explored since Milgram’s famous obedience studies [32]. Nonhuman primates have also been shown to have mental representations of social hierarchy [33] (a capacity even demonstrated in fish [34]) and are sensitive to social inequality [35].

Testing the category of social psychological threat in the present experiment is pertinent to open questions remaining from the three prior physical threat scenario studies [2, 21, 22]. For instance, Blanchard [2] argues that risk assessment can play a crucial role in detecting and analyzing threat stimuli. Risk assessment is a highly adaptive process that takes into account the type and location of the threat, as well as the escapability of the situation to predict the most optimal defense mechanism. In fact, risk assessment becomes more important when there is some degree of ambiguity in the situation [4], as is more often the case in situations of psychological social threat—a threat category we investigate here in our extension of the original Blanchard study [2]. In the psychological domain, most complexity arises from the situational context (with: peers, inferiors, or superiors; or location: work, novel setting, recreational location). We would thus expect that each of these situations creates a unique hierarchy of threat characteristics to be evaluated. Behavioral hierarchies are a prominent ethological concept: according to Tinbergen [36], an animal will enter one of a handful of broad behavioral hierarches, e.g., defense or reproduction, that then dictate further subordinate behavioral repertoires, all depending on an animal’s evaluation of the environmental context.

The situational evaluation emphasized by ethologists offers a point of contact with the human psychology literature, notably appraisal theory as articulated by Arnold [37], Lazarus [7], and Scherer [38, 39]. Appraisal theory postulates so-called “stimulus-evaluation checks”—specific dimensions upon which stimuli are sequentially or hierarchically assessed—that are used to appropriately assess context across points in time [38, 39]. For instance, first, a stimulus would be checked for relevance; if it were novel and/or (un)pleasant, it will be attended and possibly prompt initial approach or avoidance responses (e.g., pupil dilation, heart rate changes, locomotion). Once attended, the implications of the stimuli would be checked—whether they were likely to produce a consequence for the organism and the urgency with which they require a reaction. Subsequent checks relate to the organism’s coping potential for likely consequences as well as how those consequences relate to issues of normative significance such as ideas of self and social norms. Each of these hierarchical evaluations or stimulus-evaluation-checks relate to patterns of bodily, neural, and behavioral response, and can be conceptualized as a temporal unfolding of emotion [40].

The first goal of our study was to test the generalizability of dimensional factors and specific situational appraisal in guiding defensive responses across a broad range of threats. Recently, a Survival Optimization System (SOS) model has been theoretically proposed to account for cross-species threat responses [41]. A notable feature of the SOS model is that it integrates dimensional (imminent threats elicit reflexive responses) and appraisal-like accounts of threat responses. We predict that our empirical account of the ecology of human threat reactions will also highlight the relative strength of dimensional accounts in accounting for basic behavior (specifically, approach-avoidance), while situational appraisal will predict specific instantiations of approach and avoidance behaviors.

In addition to extending the range of threat scenarios, and hence the anticipated range and specificity of defensive behaviors, a second goal of our study was to then use this more comprehensive inventory of threat responses to create a generalized model for characterizing human defensive behavior toward threat. Inspired by both the appraisal theory models discussed above, Tinbergen’s behavioral hierarchies [36], and the recent SOS model [41] as well as antecedent flow-chart models (e.g., [42]), we aimed to build a hierarchical decision-tree that would accurately predict a subject’s threat response based on features of the threat stimulus. To build such a general decision tree, we aimed to sample different sources of threat, although each type of threat was only sparsely sampled by a few specific scenarios. We hypothesized that many types of defensive states—anxiety, fear, panic—could be mapped to a proximity factor similar to that in predator imminence theory [5]. While basic approach/avoidance processes might remain the same across threat domains (e.g., psychological and physical), we also expected to find differences linked to the specific demands required by certain contextually dependent types of threat [29]. We achieved our two aims of (1) contrasting ecological patterns of threat response across a broad class of threats, including psychological threats, as well as (2) organizing those patterns of threat response into a decision tree incorporating dimensional, approach-avoidance and hierarchical, appraisal-like features to eventually predict specific defensive responses.

## Materials and Methods

### Subjects

We tested 5 nonoverlapping groups of subjects over the internet as described below. The dependent measures they provided are summarized in Table 1.

**Table 1:** Dependent Measures in Experiments. All dependent measures were given for all 29 threat scenarios (cf. Table 2).

Experiment	Subject Response	Response Options	
		Physical Scenarios (n = 20)	Psychological Scenarios (n = 9)
Main; Replication (n = 85; n = 22)	Chose up to 3 top response options for each of the 29 scenarios	1. Hide	1. Hurt the other person physically
		2. Freeze, become immobilized	2. Hurt the other person verbally or yell
		3. Run away, try to escape, remove self (flight)	3. Verbal confrontation
		4. Threaten to scream or call for help	4. Avoidance or ignore the situation
		5. Yell, scream, or call for help	5. Hide or remove self from the situation
		6. Threaten to attack	6. Freeze up
		7. Attack or struggle	7. Ask for advice and/or plan a course of action
		8. Check out, approach, or investigate (risk assessment)	8. Negotiation
		9. Look for something to use as a weapon	9. Report to a higher authority
		10. Beg, plead for mercy, or negotiate	
Approach-Avoid (n = 31)	Indicated an approach-avoidance response for each of the 29 scenarios	Approach-Freeze-Avoid ratings were made using a slider on a 9-point scale	Same Approach-Freeze-Avoid scale as used for the Physical Scenarios
Factor Ratings (n = 33)	For each of the 29 scenarios, used a slider to give Low (1) to High (5) ratings for 10 descriptive factors (right)	1. Dangerousness	Same 10 factors used to characterize the Physical Scenarios
		2. Escapability	
		3. Ambiguity	
		4. Distance to threat	
		5. Presence of a hiding place	
		6. Immediacy	
		7. Ability to communicate with the threat	
		8. Ability to mitigate or change the threat	
		9. Ability to harm the threat	
		10. Ability of others to help	

#### Ethics Statement

All subjects provided informed assent to participate in research under a protocol (RA-392: “Anonymous Online Surveys of Threat Assessment”) that was approved by the Caltech Committee for the Protection of Human Subjects as Institutional Review Board exempt under Part 46.101(b)(2), “Protection of Human Subjects” of Title 45 of the U.S. Code of Federal Regulations. Instead of providing formal written consent, in our assent procedure, at the beginning of the online experiment, anonymous subjects read a description of the experiment in which they were told they were free to cease participation at any point.

#### Main experiment

88 English-speaking subjects living in the United States were recruited through Amazon’s Mechanical Turk. Subjects were paid approximately $8–10 upon the completion of the survey, and were given a maximum of 5 hours to complete the survey online. Responses from 85 (44 female) subjects (age = 33±9 years, mean±SD) were analyzed. Data from two subjects were excluded since the subjects had a diagnosis of PTSD, and a third subject was excluded because of an anxiety diagnosis and high state anxiety as measured by the State-Trait Anxiety Inventory (Spielberger, 1983). High state or trait anxiety cutoff scores were defined as 1.5 standard deviations greater than the mean score off all subjects across all 4 experiments (all but the factor rating task); cutoff scores were 58 for state anxiety and 60 for trait anxiety. Forty-four percent of subjects had a college degree or higher.

#### Scenario factor ratings

An additional independent set of 33 (17 female) American raters (age = 34±11), were recruited through Mechanical Turk to quantitatively characterize the scenarios, on a scale from 1 (low) to 5 (high), with respect to 10 pre-defined factors. The scenarios were designed in advance to vary along these dimensions; external ratings allowed us to validate and quantify variation in pre-assigned low/moderate/high ratings.

#### Replication experiment

Results from the main experiment were used to build a decision tree that predicted people’s responses to threat scenarios. To test the reliability of that decision tree, an additional set of 25 American subjects were recruited through Mechanical Turk to replicate the original threat scenario experiment. Responses from 22 (13 female) subjects (age = 33±11) were analyzed; 3 subjects were excluded for anxiety diagnoses and high trait anxiety.

#### Approach-avoidance experiment

To directly relate responses for psychological and physical threat scenarios, whose specific response options differed and thus made them impossible to compare directly in the main experiment, approach-avoidance responses to all scenarios were collected from 35 subjects. Responses from 31 (18 female) subjects (age = 33±9) were analyzed; 2 were excluded because of a diagnosis of PTSD, and a further 2 were excluded because of diagnoses of anxiety and high state and trait anxiety.

### Materials

Subjects were presented with twenty-nine scenarios in total (Table 2 and S1 Table). We designed the scenario descriptions to be relatively concise, simple, and clear. Each scenario contained an instance of one of four categories of potentially threatening situations: one that involved a natural disaster (N; 4 scenarios), an animal (A; 5 scenarios), a physical interaction with another person (P; 11 scenarios), or, in opposition to these three physically threatening categories (20 scenarios total), an interaction with another person that was more psychologically threatening (S; 9 scenarios). All scenarios included in the human physical category were directly taken from Blanchard et al. [2].

**Table 2:** Example Threat Scenarios Presented to Subjects. Each scenario is assigned a brief descriptor and label, used throughout the paper. N = Natural; A = Animal; P = Physical; S = Psychological. Full set of 29 scenarios presented in S1 Table. All Physical scenarios taken from [2].

Descriptor	Scenario	Label
Hurricane, 10 min	Imagine you are living in New York City, and you hear on the news that a new hurricane is arriving in 10 minutes. It is going to hit the city any moment now. This one is going to be even bigger than Hurricane Sandy, and no one knows what to make of it.	N1
Bear, 50 yds	You are camping in the mountains. You go out by yourself to take a walk, and you suddenly see a bear approaching from 50 yards away.	A1
Grab	You are alone as you exit an empty campus building late one night. Just as you get outside you feel a hand grab your arm.	P8
Rumor	Recently, you have noticed that one of your co-workers has been talking behind your back at work. He/she has been spreading rumors, and seems to drop negative remarks about you to your immediate boss as well.	S4

These scenarios were designed to vary along 10 different factors (Table 1). The first five factors were derived from Blanchard et al. [2], and we included additional factors to reflect our expanded set of scenarios (e.g., ability to communicate to capture human vs. animal scenarios; ability to mitigate to capture elements of social support and social hierarchy).

In order to rate each of these scenarios along the above dimensions, independent raters were each presented with 10 randomly selected scenarios from the set of 29 and asked to rate each of those scenarios for each factor (dangerousness, escapability, etc.) on a scale of 1 (low) to 5 (high) for all 10 factors. Through random assignment, each scenario was rated by at least 8 and up to 15 individuals (mean = 11.4).

### Procedure

#### Main and Replication Experiment

Subjects were asked to read each scenario and indicate their most likely first-responses. Subjects also had the option to choose up to two additional options. If they had selected multiple options, subjects were asked to rank their responses from 1 (most likely) to 3 (least likely); here we analyze only the data from the top response option. The psychological scenarios were given a separate category of response options. There were 10 response options for the physical scenarios (natural, animal, and human) and 9 for the psychological scenarios (Table 1).

#### Scenario Factor Ratings

Subjects were randomly presented 10 of the 29 scenarios, and asked to use a sliding scale to provide factor ratings for each scenario on a scale from 1 (low) to 5 (high), with respect to all 10 of our pre-defined factors (cf. Table 2). The starting position of the sliders was randomized. Because subjects were only presented 10 of the 29 scenarios, factor ratings for each scenario were provided by a subset of the 33 subjects. A minimum of 8 and maximum of 15 subjects rated each scenario, with 21 of the 29 (72%) scenarios being rated by at least 10 subjects.

#### Approach-Avoidance Experiment

**Fig 1:**
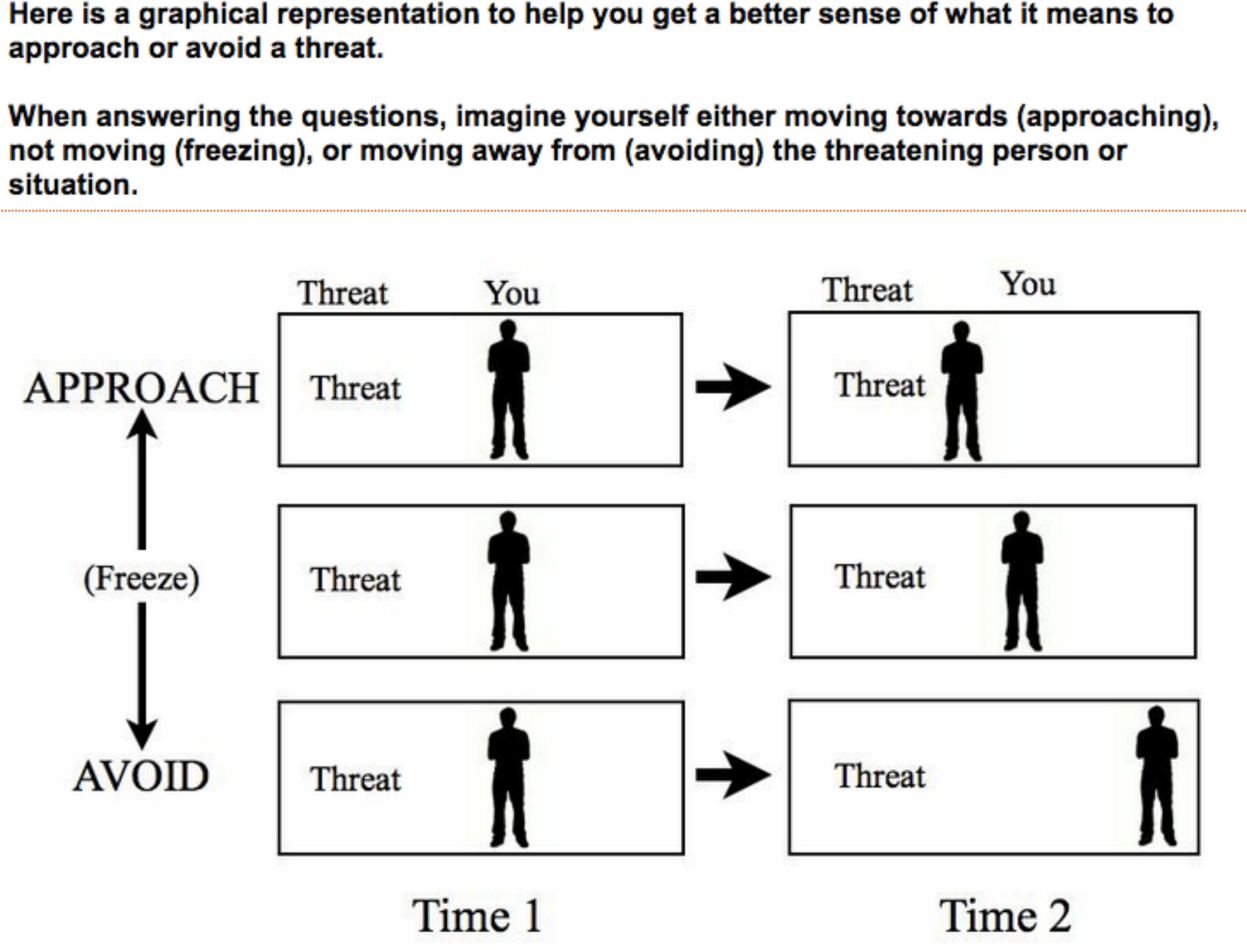
Approach/avoid instructions. Subjects (n = 31) in the approach-avoid experiment viewed these instructions, which made explicit that approach-avoidance ratings related to taxis relative to the source of threat.

Subjects were instructed to read each of the 29 scenarios and imagine how they would respond to the threat in terms of approach/avoidance. An illustration (Fig 1) explained the concept of approaching/freezing/avoiding a threat. Subjects indicated their response on a 9-point slider, which began in the middle of the range. Subjects were asked to imagine themselves as the slider moving either toward (left) or away (right) from the threat.

### Analyses

#### Factor ratings

Means and standard deviations are reported for the independent ratings along 10 factors, and were used to characterize each scenario. The averaged independent ratings ranging from 1.00 to 5.00 were normalized across all scenarios (Normalized Scenario Score = (Scenario Score—Minimum Factor Score)/(Maximum Factor Score—Minimum Factor Score)) such that the lowest rated scenario for a factor received a score of 0, and the highest rated scenario received a score of 1.

#### Factor-specific response correlations

To quantify relationships between each of these rated factors and the set of defensive behaviors, we calculated Pearson product-moment correlations for every possible factor-response pairing. The first vector in this correlation consisted of the mean factor rating given to each scenario; the second vector consisted of the proportion of subjects who chose a response option as their first choice for each scenario. Because response options differed between physical and psychological scenarios, all analyses were conducted separately for those scenario categories. To visualize patterns of correlations, correlation coefficients were reported in heatmaps with factors and response options organized along rough imminence and approach-avoidance continua, respectively.

#### Factor-approach/avoid response correlations

To directly relate responses to psychological and physical threat scenarios, the same correlational analysis completed for specific response options was completed according to the proportion of subjects who chose approach-avoid ratings corresponding to categorical approach/freeze/avoid responses.

#### Gender differences

The above analyses were completed for males and females separately, as well as together. While minor differences were found between male and females’ first choice defensive behaviors for some scenarios, as these differences mirrored prior findings [2] (Table 3) and did not reflect our primary interest, males and females are consequently pooled in the results with specific differences noted only as they arise.

**Table 3:** Gender Differences. Comparison of male and female top response options in scenarios for which their first responses differed. When applicable, these differences are compared to prior results [2] in the comments column. While the top response option in scenario P10 did not differ between males and females, the scenario is reported since Blanchard [2] had observed a gender effect. * Denotes a tie between first-choice response options.

Scenario	Male Top Responses (n = 41)	Female Top Responses (n = 44)	Comments
P2, Elevator	1. Attack or struggle	1. Yell or scream	Blanchard found the same first choices.
	2. Yell or scream	2. Attack or struggle	First and second choices switched by gender.
P8, Grab	1. Attack or struggle*	1. Yell or scream	Blanchard found the same first choices.
	1. Risk-assessment*	2. Risk-assessment	Top three choices the same across genders.
	2. Yell or scream	3. Attack or struggle	
P10, Phone	1. Look for a weapon*	1. Look for a weapon	Observed no differences in first response
	1. Risk-assessment*	2. Yell or scream	Top three choices the same across genders.
	2. Yell or scream	3. Risk-assessment	Blanchard’s first female response was hide.
N2, Hurricane	1. Flight	1. Risk-assessment	Comparison to Blanchard not applicable.
	2. Risk-assessment	2. Flight	First and second choices switched by gender.
S1, Blackmail	1. Report to authority	1. Verbal confrontation	Comparison to Blanchard not applicable.
	2. Verbal confrontation	2. Report to authority	First and second choices switched by gender.

#### Single approach-avoid score

A single approach-avoidance score across subjects’ ratings was derived for each scenario, with more positive scores indicating approach, more negative avoidance, and those close to zero either indifference or freezing. To construct this score, first, approach, freeze, and avoidance scores were calculated for each scenario. These scores were the proportion of subjects choosing categorical approach (ratings of 1, 2, 3, 4)/freeze (ratings of 5)/avoid (ratings of 6, 7, 8, 9) for each scenario, with the proportion of subjects choosing an approach or avoidance rating weighted by subjects’ median approach or avoidance score. All categorical approach/freeze/avoid scores were rescaled on a 0 to 1 interval. Then, a single approach-avoidance score took the signed absolute value of the difference between the rescaled approach and avoidance scores, and penalized it by subtracting the magnitude of the rescaled freeze score for that scenario, such that the single approach-avoid score for scenarios that had larger freeze scores were closer to zero.

#### Decision tree

A descriptive decision tree that predicted responses to threat scenarios based on features of those scenarios was created through a multi-step process.

The first major step was describing convergence or divergence between physical and psychological scenarios. Two analyses guided this step. First, to test our hypothesis that psychological and physical threats are characteristically distinct, we calculated the dissimilarity between all pairs of scenarios based on the factor ratings of those scenarios, using the correlation distance measure in Matlab’s pdist function. These pairwise scenario dissimilarities were then visualized both (1) as a dissimilarity matrix heatmap, organized by scenario type—animal, natural, human physical, and psychological—and (2) according to multidimensional scaling of the dissimilarity distances between each scenario, to determine whether physical and psychological threats cluster separately. The results of this analysis partially guided early splitting of physical and psychological threat.

Secondly, we sought to determine whether basic approach/avoidance behavior to psychological and physical threats diverged according to any factors. To do this, the single approach-avoidance scores were correlated with factor scores to guide construction of the beginning of the decision tree.

After forming the top branches of the tree, which predicted primary approach/avoidance responses, we extended the tree to predict appraisal-related specific responses (e.g., risk assessment, attack, verbal confrontation, etc.). This portion of the tree was constructed by summarizing how scenarios with the same most popular response options varied with respect to factor ratings. In organizing these nodes of the decision tree, priority was given to explanatory factors that (1) clustered consistently to yield a common top response option (close ties were allowed), and that (2) made ecological sense or adhered to a priori hypotheses (e.g., about the importance of communication, threat imminence, etcetera). While there were no overall gender differences in basic approach-avoidance behavior, specific responses to scenarios sometimes varied with gender; therefore, when appropriate, gender was used as a late node in the tree.

After construction, we tested the predictive success of the tree by calculating the proportion of each individual subjects’ responses that were correctly predicted for both the original and replication groups and comparing this prediction to chance performance of around 10% (10 vs. 9 specific response options for physical and psychological threat, respectively). Importantly, the decision tree was derived based only on the original group’s data, and thus the replication group was an independent set of data on which to test.

## Results

### Independent Factor Ratings

The scenarios were designed to vary along the 10 specified factors. Externally validating our construction, low (0)/moderate (0.5)/high (1) pre-determined factor ratings (assigned by the experimenters when constructing the scenarios) correlated significantly (*p*<0.001) with all measured factor ratings (scale 1 to 5, measured in the factor-rating experiment (n = 33)).

Across all four threat categories (physical, natural, animal, psychological), scenarios spanned the range of subjects’ (n = 33) raw factor ratings well (Table 4). The physical scenarios had the highest ratings for escapability, ambiguity, and ability to harm (Table 5); these ratings were similar to the previously reported ratings for these scenarios [2].The natural scenarios were rated as especially dangerous and low in ability to communicate. The animal scenarios were rated high in immediacy, and, like the natural scenarios, low in ability to communicate. The psychological scenarios were rated high in ability to mitigate and ability of others to help as well as the ability to communicate.

**Table 4:** Scenario Factor Ratings. Columns demonstrate the range of raw ratings by factor, with lowest and highest rated scenarios listed. Histograms (S1 Fig) show the number of scenarios that received an average rating corresponding to a score of 1 (low) to 5 (high).

**Factor**	**Low**	**Low scenario**	**High**	**High Scenario**
Dangerousness	1.00	Party; bar	4.94	Bear 1yd
Escapability	1.70	Hurricane 10 min	3.44	Acquaintance
Ambiguity	1.25	Boss	4.50	Acquaintance
Distance	1.08	Grab	3.14	Tornado 24hr
Availability of a hiding place	1.25	Bear 1yd	3.46	Whisper
Immediacy	1.95	Party	4.92	Elevator
Ability to communicate	1.00	Bomb; Hurricane; Tornado	4.53	Blackmail face
Ability to mitigate	1.42	Hurricane 24hr	4.15	Political
Ability to harm	1.00	Hurricane	3.89	Acquaintance
Ability of others to help	1.60	Noise	3.58	Homophobic
**Factor**	**Low**	**Low scenario**	**High**	**High Scenario**
Dangerousness	1.00	Party; Bar	4.94	Bear 1yd
Escapability	1.70	Hurricane 10 min	3.44	Acquaintance
Ambiguity	1.25	Boss	4.50	Acquaintance
Distance	1.08	Grab	3.14	Tornado 24hr
Availability of a hiding place	1.25	Bear 1yd	3.46	Whisper
Immediacy	1.95	Party	4.92	Elevator
Ability to communicate	1.00	Bomb; Hurricane; Tornado	4.53	Blackmail face
Ability to mitigate	1.42	Hurricane 24hr	4.15	Political
Ability to harm	1.00	Hurricane	3.89	Acquaintance
Ability of others to help	1.60	Noise	3.58	Homophobic

**Table 5:** Category Factor Ratings. Normalized factor ratings (Mean ± SE) by scenario category.

Factor	Natural (n = 4)	Animal (n = 5)	Physical (n = 11)	Psychological (n = 9)
Dangerousness	0.89 ± 0.05	0.84 ± 0.05	0.72 ± 0.04	0.26 ± 0.08
Escapability	0.45 ± 0.13	0.53 ± 0.14	0.69 ± 0.07	0.51 ± 0.07
Ambiguity	0.41 ± 0.04	0.42 ± 0.14	0.56 ± 0.08	0.28 ± 0.06
Distance	0.62 ± 0.16	0.51 ± 0.14	0.36 ± 0.08	0.60 ± 0.09
Availability of a hiding place	0.69 ± 0.08	0.19 ± 0.08	0.45 ± 0.11	0.28 ± 0.09
Immediacy	0.68 ± 0.10	0.76 ± 0.07	0.75 ± 0.05	0.38 ± 0.08
Ability to communicate	0.00 ± 0.00	0.04 ± 0.01	0.51 ± 0.08	0.84 ± 0.05
Ability to mitigate	0.22 ± 0.11	0.16 ± 0.04	0.53 ± 0.06	0.65 ± 0.07
Ability to harm	0.00 ± 0.00	0.23 ± 0.03	0.65 ± 0.06	0.47 ± 0.08
Ability of others to help	0.40 ± 0.11	0.22 ± 0.10	0.37 ± 0.05	0.57 ± 0.09

### Specific situational factors elicit specific behaviors

**Fig 2:**
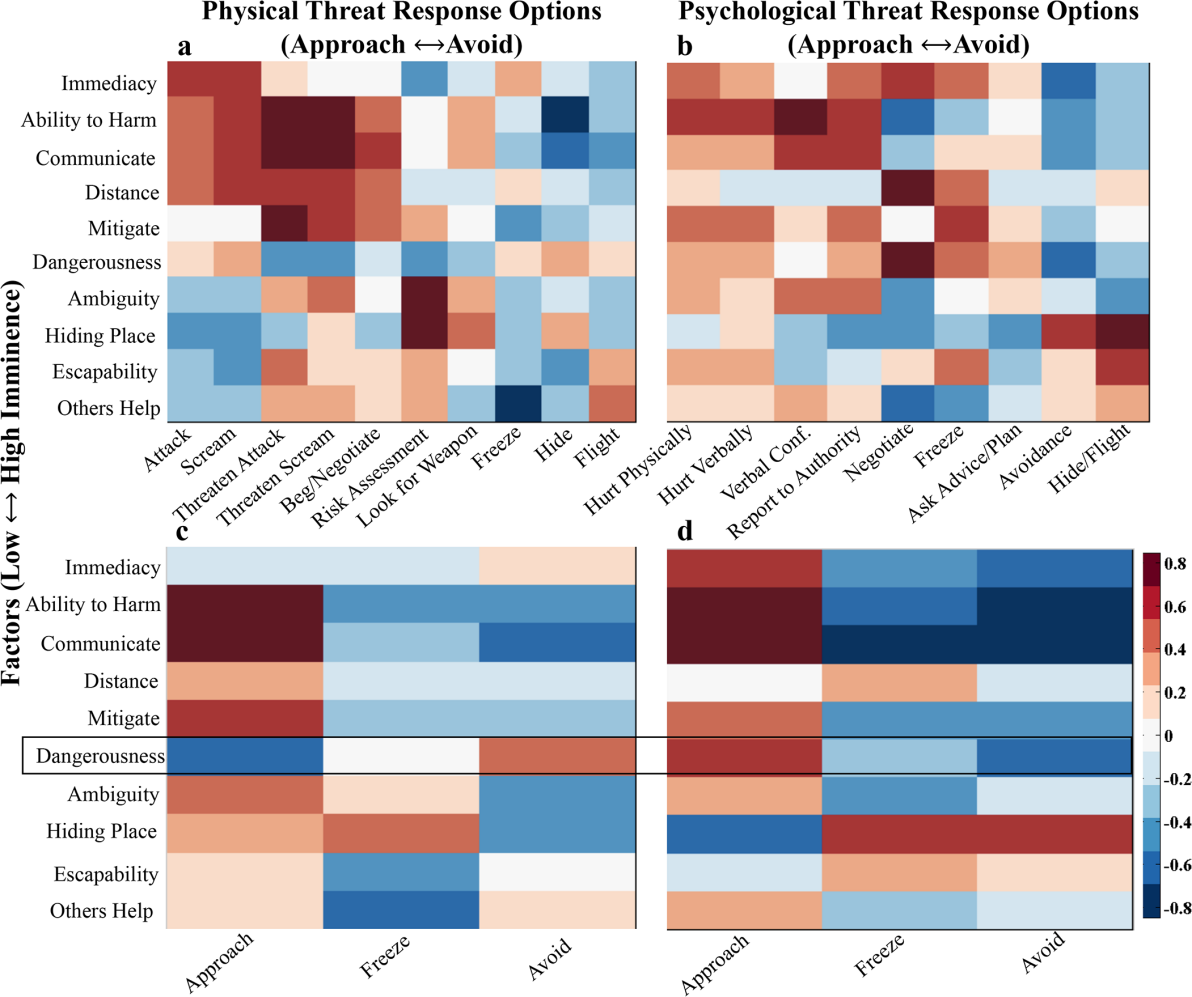
Factor-Response Option Correlations. Heatmap of correlation coefficients from Pearson’s correlations between mean factor ratings and specific defensive behaviors (**a,b**) or approach/freeze/avoid (**c,d**) for physical (left) and psychological (right) threat scenarios. Row-wise factors organized along an approximate low to high imminence continuum. Column-wise response options organized along an approximate approach-avoidance continuum. Original distance scores were reversed to far-to-near to in accordance with the imminence continuum.

Correlations between the mean factor ratings and the proportion of subjects endorsing defensive behaviors were calculated to determine whether human defensive behavior could be predicted by certain situational factors across categories of threat. All non-psychological scenarios (natural, animal, and human physical) were combined in the correlations (Fig 2A), with results for psychological scenarios reported separately (Fig 2B).

Many of the highest correlations were predictable: in the physical scenarios, the ability to communicate with the source of threat was strongly correlated with threatening to attack or threatening to scream; in the psychological scenarios, the presence of a hiding place was strongly correlated with hiding. A global pattern existed, such that imminent threats tended to be approached. For example, threats that were high in immediacy, dangerousness (all scenarios were dangerous, but some more so than others), ability to harm, or proximity were positively correlated with responses that required approaching the source of threat, like attacking, screaming, or threatening to do so. On the other hand, threatening scenarios that were escapable, ambiguous, or had a hiding place available were negatively correlated with those approaching actions and positively correlated with avoidant actions including risk-assessment and hiding. This pattern was strongest for the most imminent threats, suggesting a more rigid and restricted set of response patterns to these scenarios.

### Comparison of Psychological and Physical Threats with Direct Approach-Avoid Ratings

Direct comparisons of responses to psychological and physical threats as a function of factor ratings were made using approach-avoidance ratings. Correlations between the proportion of subjects choosing to approach, freeze, or avoid a threat and the factor rating of a threat elicited a similar pattern observed for specific responses, whereby more imminent threats tended to be approached (Fig 2C and 2D). This pattern held for both physical and psychological threats, with a notable exception for the factor of dangerousness (the magnitude of the threat): dangerous physical threats were avoided, and dangerous psychological threats approached.

### Decision Tree

**Fig 3:**
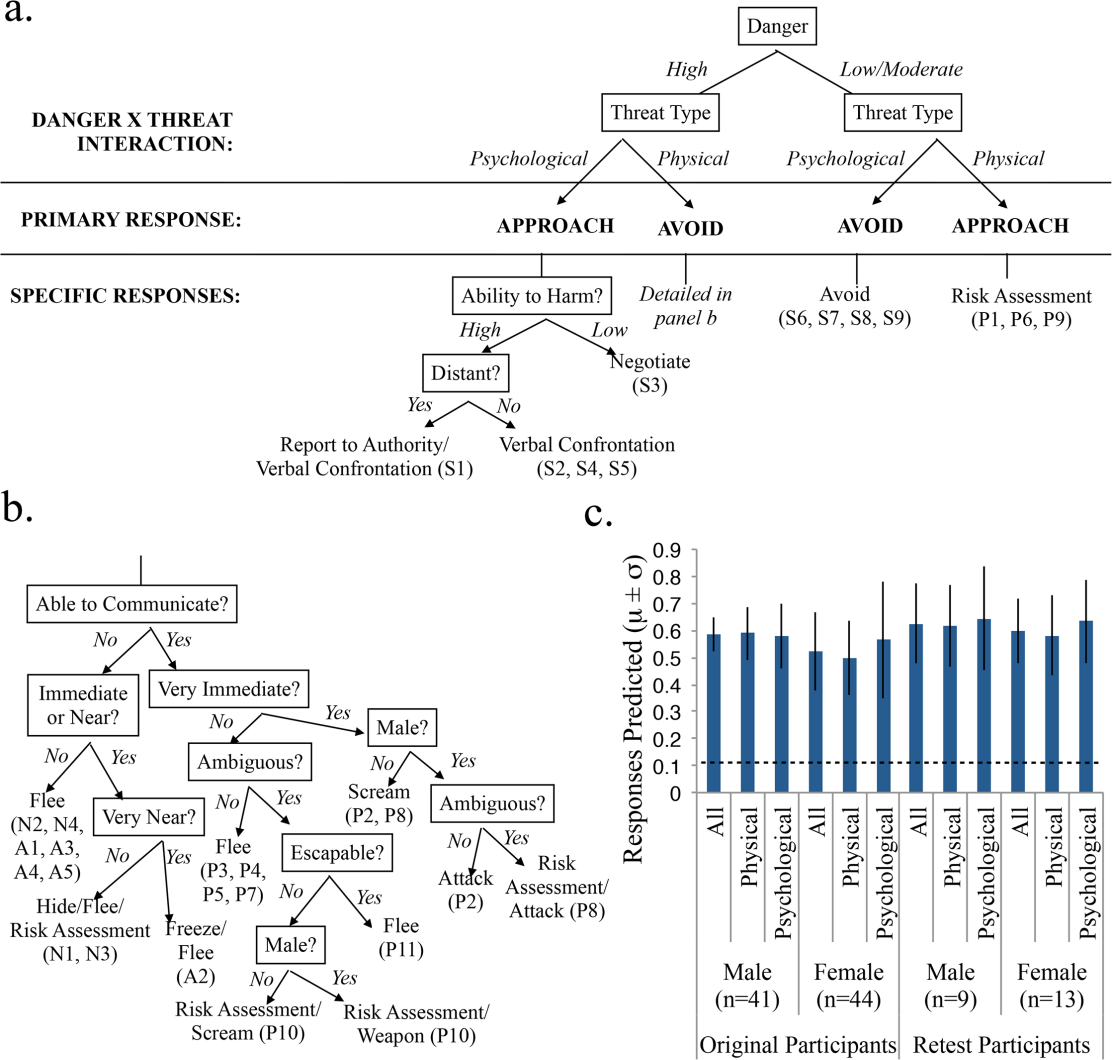
Decision Tree for Defensive Behaviors to Threatening Scenarios. **Panels a, b.** Decision tree predicting the defensive behavior chosen by the majority of subjects based on characteristics of that threat scenario. The tree consists of four main branches, with primary approach/avoid responses predicted by the 2x2 interaction of danger (high, moderate) and threat type (psychological, physical threat). Appraisal of factors along further nodes predicts specific defensive responses for each scenario, denoted by the scenario labels used in Table 2. Where appropriate, gender differences are noted. The tree successfully predicts the group majority decisions of both original subjects (n = 85) and separate replication study subjects (n = 22) for all scenarios. **Panel c.** The average proportion of original and replication study subjects’ first responses correctly predicted for all scenarios (n = 29), physical scenarios (n = 20), and psychological scenarios (n = 9). Male and female performance reported separately. Dashed line around 0.12 (All: 0.128; Physical: 0.130; Psychological: 0.123) represents chance performance.

**Fig 4:**
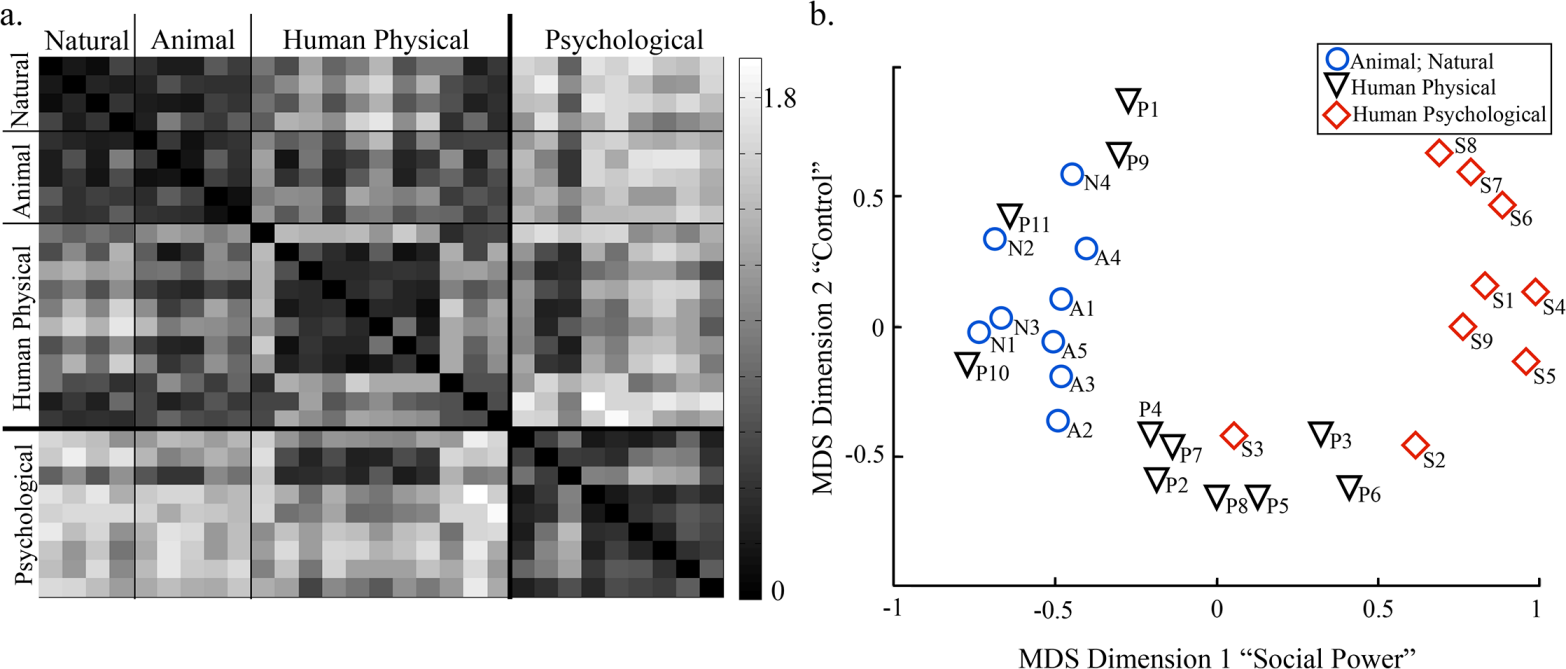
Clustering of Scenario Categories Based on Factor Ratings. **Panel a.** Heatmap of scenario factor ratings dissimilarity matrix. Dissimilarity scores (legend right of heatmap) represent the distance between pairs of scenarios, calculated as one minus the sample correlation between the ten factor ratings for each pairwise scenario comparison. Scenario labels indicated on top and left of heatmap, with individual scenarios denoted by each row/column (i.e., 4 “Natural” scenarios: N1, N2, N3, N4). Black diagonal indicates scenarios are minimally dissimilar to themselves; dark clusters indicate within-category scenarios are most similar according to factor ratings. Psychological scenarios are most distinct from the other categories. Within category similarity exceptions exist, e.g. P1, P9, P11 and S3. **Panel b.** Two-dimensional multidimensional scaling (MDS) of Euclidean distance between scenarios based on factor-rating dissimilarity scores. Human psychological threat scenarios (red stars) mostly clustered separately from physical threats (blue circles: animal and natural threats; black triangles: human physical). A scree plot of stress by MDS dimensions justified the use of 2 dimensions, which had a stress of 0.140. While the primary value of our MDS analysis is as a visualization of the similarity space of scenario factor ratings, we cautiously suggest that the first MDS dimension, positively related to ability to mitigate (r = 0.68) and to communicate (r = 0.90) with the threat, while inversely related to dangerousness (r = -0.88) and immediacy (r = -0.63), related to “social power” or the threatened individual’s ability to communicate with and influence the threat. Meanwhile, the second MDS dimension, inversely related to immediacy (r = -0.68), and positively related to distance (r = 0.74) and the presence of a hiding place (r = 0.75) captured the threatened individual’s ability to thwart the threat and “control” the situation.

A data-driven decision tree (Fig 3) that predicted a person’s choice of defensive behavior for all scenarios summarized the most deterministic relationships between scenario factors and defensive behaviors. The construction of this tree was based entirely on data from the original sample (n = 85), and was later tested on data from the replication sample (n = 22). Partially supported from a clustering of scenarios based on patterns of factor ratings (Fig 4), the beginning of the tree separated psychological from physical threats.

The primary difference in reactions to psychological and physical threat is concisely summarized by differences in the interaction between dangerousness and basic approach/avoidance responses. Correlations between the single approach-avoidance scores and factor ratings for psychological dangerousness and physical dangerousness separately showed a significant positive correlation with psychological dangerousness (*r* = 0.68, *p* = 0.000) and a significant negative correlation with physical dangerousness (*r* = -0.57, *p* = 0.001). In other words, dangerous psychological scenarios were approached, and dangerous physical scenarios were avoided. This pattern held for both females (psychological: *r* = 0.50, *p* = 0.006; physical: *r* = -0.52, *p* = 0.004) and males (psychological: *r* = 0.49, *p* = 0.007; physical: *r* = -0.39, *p* = 0.037).

The pattern of this interaction separated the four major branches of the decision tree into the following branches: (1) high and (2) moderate danger physical threats, and (3) high and (4) moderate danger psychological threats. The primary response tendency for (1) and (3) was avoidance; types (2) and (4) were primarily approached.

The latter portion of the tree predicted specific responses. Traversing down the tree, an assessment was made at each node, related to a specific factor, to ultimately predict an action. The dominant response to low-threat psychological scenarios was simply avoidance; low-threat physical scenarios were predominately assessed for risk.

The path to specific responses for high danger scenarios was more complicated. The ability to communicate with the source of threat split human from non-human scenarios early in the tree. Animals and natural disasters, which could not be communicated with, and which were also physically difficult or impossible to fight, prompted flight, unless they were quite close, in which case risk assessment or hiding, and freezing (for especially imminent threats) were employed. For human physical threats, immediacy, ambiguity, escapability, and gender interacted to predict responses. For psychological threats, verbal confrontation was a popular approach option. Meanwhile, negotiation was employed in a scenario involving a boss where there was low ability to harm the source of threat. For more distant threats, reporting to an authority was a popular response option.

This decision tree correctly predicted the most popular response for all 29 scenarios in both the original and replication groups and performed much better than chance (about 12% since some scenarios allowed more than one valid response option) at predicting individual subjects’ responses for both the original and replication groups (Fig 3C).

## Discussion

### Main Findings

#### Correlations

Whilst using a new methodology (internet collection of scenario responses), we replicated prior findings that human reactions to human physical threats mirror patterns of defensive responses observed in rodents [2, 21, 22] (Table 6). We extended this to show that similar patterns exist for defensive responses to non-human physical threats (natural disasters and animals), as well as social psychological threat, with some notable differences.

**Table 6:** Comparison of correlations coefficients between defensive behaviors and scenario characteristics obtained in 4 studies. Comparison between Blanchard [2], Perkins and Corr [22], and Shuhama [21] reproduced from Blanchard [4]. The first 3 studies (3 leftmost columns) used Blanchard’s original 12 physically threatening scenarios and report male (top) and female (bottom) correlation values separately. In the present study (4 rightmost columns), the 20 physical scenarios included 11 of the original physically threatening scenarios, along with 4 natural disaster and 5 animal scenarios. For the 9 psychological scenarios, one to two comparable defensive response options are reported. V.C. = verbal confrontation. *p<0.05, **p<0.01, ***p<0.001, n.s. = not significant with *p*<0.05; *p*-values not reported in Shuhama et al. [21].

Defensive behavior/factor	Blanchard (Hawaii)	Perkins & Corr (Wales)	Shuhama (Brazil)	Original (USA)	Animal; Natural (USA)	Physical (USA)	Psychological (USA)
**Risk assessment/ ambiguity**	0.89**	0.89**	**0.91**	0.93***	**0.08**	0.62**	**0.13 (plan)**
	0.86**	0.85**	**0.88**				
**Flight/ ambiguity**	**-0.50**	**-0.56**	**-0.69**	**-0.50**	0.73*	**-0.20**	**-0.48 (hide)**
	**-0.63**	-0.59*	**-0.61**				**-0.13 (avoid)**
**Defensive attack/ ambiguity**	**-0.53**	**-0.54**	**n.s.**	**-0.42**	**-0.50**	**-0.23**	**0.41 (V.C.)**
	**-0.23**	**-0.44**	**n.s.**				
**Flight/ escapability**	**0.10**	**0.12**	**n.s.**	**0.35**	0.81**	**0.33**	**0.66 (hide)**
	**0.04**	**0.10**	**n.s.**				**0.23 (avoid)**
**Defensive attack/ escapability**	-0.76*	-0.87**	**-0.76**	-0.67*	-0.60*	**-0.30**	**-0.38 (V.C.)**
	-0.65*	-0.89**	**n.s.**				
**Defensive attack/distance**	-0.59*	-0.62*	**n.s.**	-0.47*	-0.71*	**-0.43**	-0.72* (negotiate)
	-0.64*	-0.69*	**-0.69**				
**Hiding/hiding place**	0.59*	**0.33**	**0.61**	0.81**	**0.44**	**0.29**	0.84** (hide)
	0.63*	**0.30**	**0.59**				

#### Decision Tree

Features of the threat scenarios determined behavioral responses; these patterns were summarized in a decision tree that successfully predicted scenario responses for the original subject group as well as generalized to a replication sample. This tree demonstrated two processes at play in threat reactions: (1) basic approach/avoidance behavior and (2) situational appraisal. The first set of processes distinguished psychological from physical threats based on a single factor—the magnitude of the threat: more dangerous physical threats were avoided while more dangerous psychological ones were approached. Subsequently, appraisal of further factors determined the best specific response for a particular threat scenario.

#### Imminence Framework

While our decision tree splits psychological from physical threat as a function of dangerousness, a general dimensional framework across both threat types emerges from the pattern of correlations between situational factors and favored defensive behaviors. Looking across the heatmap columns in Fig 2, behavioral responses can be organized along an approach-avoidance continuum, with freezing in the middle. Traversing the rows of Fig 2, situational factors exist on an imminence continuum. As a threatening situation becomes more imminent—immediate, close, and dangerous—attack responses are chosen; as the immediate threat wanes, avoidant behaviors, which are less costly to the organism, are adopted. Approaching actions (e.g., attack, negotiate) are only taken when an organism is pressed by imminent threat, with the exception of imminent but escapable threats, which are avoided. This structure mirrors a previously described pattern [43] whereby regardless of whether a predator or social conspecific posed a threat, imminent threats (e.g., a cat to a rodent or a dominant rhesus macaque to another rhesus macaque) evoked fast reflexive behaviors (escape/freeze/defensive aggression) while more distant threats (e.g., cat odor or a photo of a dominant rhesus macaque) are cautiously explored. This pattern of imminent threats evoking fast, reflexive responses was recently emphasized in the theoretical Survival Optimization System (SOS) model [41]. Likewise, in conditioned fear paradigms, a conditioned stimulus is an imminent predictor of an aversive stimulus and elicits a prompt response. Our findings agree with the pattern of imminent threats eliciting rapid responses: across domains, imminent threats provoke fast, reflexive actions while more distal threats permit exploration. These generic patterns observed across threat categories likely reflect conserved adaptive mechanisms that evolved to cope with physical predator threat and that were subsequently co-opted for coping with social/psychological threats.

The importance of threat imminence to explaining defensive behaviors in this two-dimensional manner is in line with prior work on predator imminence [5, 8]. Indeed, the brain is in fact sensitive to the literal distance to a threat: brain activity shifts from the ventromedial prefrontal cortex to the periaqueductal grey as the imminence of a virtual predator in an fMRI experiment is increased [44]. It is even the case that activation in the amygdala discriminates the directionality of a threat—a tarantula—either towards or away from a subject, regardless of actual distance. This ability of the brain to monitor many dimensions of threat provides direct neurobiological evidence that we “fractionate” basic fear into component mechanisms [45]. A major challenge for the future will be to map such neural components as revealed with fMRI [45] or cellular techniques [29], onto the appraisal-like components we identified in our decision tree (Fig 3). While our stimuli here were designed to be concise for this behavioral experiment, it would be useful to design future stimuli that could also be used in fMRI tasks; such stimuli would need to control more stringently for a host of lexical and semantic confounds including length, word frequency, readability, concreteness, and arousal, all of which were not controlled for in our small sample of stimuli.

#### Interaction Caveat

An important caveat to all these dimensional analyses is that situational factors interact. Therefore, it is important to be mindful of the entire context when assessing a behavior in any species. For example, it is the interaction between imminence and perceived magnitude of the danger that explains why imminent psychological threats are only reflexively approached (defensive aggression), while escape is a popular reflexive response for (escapable) imminent physical threats. Notably, while flight was a common behavioral response and was predicted well by our decision tree (cf. Fig 3B), it did not strongly correlate with any individual physical threat features (cf. Fig 2A), likely because of the interaction between factors. The concern about careful contextual analysis extends to comparative animal research: different tests can differentiate diverse anxiety phenotypes in non-human primate models for clinical comparisons to humans. However, these tests often do not correlate well with observed diagnostic behaviors, likely exhibiting context dependency which is not generalizable across tests [46].

#### Study Limitations

This study does not directly measure real threat behavior in humans, but rather ratings about hypothetical scenarios. Therefore, one could argue that the behavior captured here relates to an intuitive, culturally learned “folk” knowledge of how one ought to respond to a threatening situation. However, it is compelling that the patterns described here in humans from reactions to hypothetical threat scenarios, in fact relate well to patterns observed in the actual behavior of rodents, as also seen in three other studies using the same methods [4]. As such, it is our assumption that our data reflect the actual behavioral structure of threat. However, it is clear that future experiments should attempt to (1) observe humans’ responses in actual threatening encounters in an observational (but non-experimental) context, (2) observe behavioral responses in an experimentally-controlled virtual-reality type of experiment in which subjects “experience” a threat but are not placed in danger, and (3) record implicit measures including changes in autonomic arousal, body sway/freeze, and emotional expression in response to these stimuli.

Additionally, it should be noted that additional features could be added to our model, for example nodes determining whether both the potential attacker and threatened individual are aware of the threatening situation [42]. All our scenarios involved an established threat situation, but future work expanding the approach we developed should also incorporate evaluations antecedent to this point in the threat evaluation process.

### What’s Different for Non-Human Physical and Human Psychological Threats?

#### Non-Human Threats

In contrast to the results from human physical threat, in our correlation analysis, there was a weaker positive relationship between risk assessment and ambiguity for animals and natural disasters (cf. Table 6). Additionally, for these two physical threat types, the relationship between flight and ambiguity reversed from negative to positive, and the positive relationship between flight and escapability was stronger. In the decision tree, these threats were never approached. These changes are likely a function of the increased danger and decreased ability to communicate with or mitigate the source of threat in animal and natural scenarios as compared with human physical scenarios—in such situations escape is prioritized.

#### Psychological Threats

While similar to human and non-human physical threats in our dimensional analysis, psychological threats were the most distinctive threat category, requiring unique specific response options. Empirically supporting our hypothesis that while defensive reactions to all threat types draw on similar processes, psychological threats are qualitatively different. Factor ratings (Fig 4) and approach-danger tendencies distinguished psychological from physical threats. Specific differences for psychological threat may arise from two main differences: the timescale of the threat and the specific type of harm inflicted.

Psychological and physical threats can be continuously mapped onto a dimension of temporal immediacy: psychological threat decouples an immediate physical threat from the cues that signal it. On an interesting side note, while unconditioned fear stimuli are directly linked to physical threat, conditioned fear stimuli are separable from actual physical threat and therefore similar in nature to our psychological threats. Tautologically, in our study, imminent psychological threats were dangerous *because* they were imminent, could not be avoided, and required a rapid response. Typically, psychological threats and reactions to them unfold more slowly over time, allowing individuals to gather information and plan an optimal response, often drawing on the advice and help of others. In our most immediate/dangerous psychological scenarios, these options were not available.

In humans, there can then be yet another layer to psychological threat that is something like "symbolic threat": e.g., blackmail, where there is no physical threat at all, but instead relevance to factors such as social reputation. Other species show such “psychological" threat to some degree, related to social rank and social ostracism, one of the most potent social threats [47]. In other species, these concerns relate more directly to physical concerns (access to food, protection, etc. with strong social bonds even increasing longevity in baboons [48]), while humans’ worries about social reputation and social exclusion have less immediate physical ramifications (although admittedly, status relates to physical outcomes). Nevertheless, human fMRI studies suggest that aspects of social threat (social exclusion) activate regions that overlap with those activated by physical threats (physical pain) [49]. The two types of pain share common pathways across several species; this evolutionary overlap has been attributed to physical pain mechanisms being used to prompt appropriate defensive reactions for social threats to inclusion [50]. Indeed, some social psychological threats (angry faces) prime defensive bodily reactions, including freezing [51].

One notable exception to the observation that behavioral patterns to psychological threat mirror those to physical threat occurred in the case of ambiguous scenarios: defensive attack was not chosen in ambiguous physical scenarios, while verbal confrontation, which is analogous to attacking, was chosen in ambiguous psychological threat scenarios. In the psychological case, it seems that the cost of confrontation is not as high as in the physical case, where attack could likely result in bodily harm. Instead, the psychological form of attack—verbal confrontation—might even garner clarification of the ambiguous situation.

### Future Directions

Three observations about our decision tree relate to future directions. Moving beyond Blanchard’s [2] correlational approach, the decision tree allowed us to recapture the complex interaction of situational factors in guiding threat responses. Hierarchically organized, our decision tree emphasized (1) the importance of stimulus category (early branches separate psychological from physical threat, and then physical threat in which another human is or is not present); (2) that certain factors are more relevant and processed earlier (e.g., danger and immediacy are assessed early because highly imminent situations require immediate action); (3) appraisal occurs at each node, and must integrate information from the prior path traveled to reach that node. The length of a path traversed is relevant: throughout the tree, the general principle of attacking/actively responding to imminent risk and retreating/avoiding in less imminent situations holds constant across situational factors and categories of threat and fewer appraisal nodes are traversed for imminent than less-imminent threats. Each of these three observations relate to other findings and future directions.

#### Neural support

First, the importance of stimulus category raises the key question of what neural support exists for psychological theories. While common pathways have been discussed, and are evolutionarily efficient, some separate processing of social threat is supported by the finding that different hypothalamic circuits exist for predatory and social fear [29]. A neural approach may also answer the open question of to what degree appraisals are automatic or controlled (deliberative); different appraisals may participate in different circuits, with varying degrees of automaticity [52].

#### Appraisal theory and relevant factors

Non-human primates appear capable of behavior similar to situational appraisal. For example, woolly monkeys learned to react differently to three types of human intruders; learning when it is appropriate to launch a defensive response to humans’ presence saves energy, leaving time for foraging [53].

That certain factors have priority for appraisals can be related to the sequential nature of stimulus-evaluation checks postulated by some appraisal theories [39, 54]. Interestingly, the enhancement of visual and olfactory sensory acquisition by fear expression [55] relates to the cumulative nature of the appraisal process: being afraid involves gathering and assessing information about the source of threat. Appraisal theory identifies pertinent stimulus attributes. For instance, Scherer [38] proposes 4 broad sets of such stimulus-evaluation checks that assess, in sequence: personal relevance of the stimulus; evaluation of how it affects well-being; coping potential; and normative significance. While our limited and in general psychologically simple set of scenarios was not designed to probe psychological appraisal theories, they share with such theories the need for a prioritized and integrative structure in how their threat is evaluated. Hierarchical assessment (cf. Tinbergen, 1951) and the contextual nature of that assessment are shared features of Scherer’s component-process model and our decision tree. Appraisal theory is concerned with emotional states [40], which may elicit a behavior, but need not do so. Meanwhile, our decision tree focuses on the outcome of an emotional state, while remaining relatively agnostic about that state (neither we nor Scherer restrict these states to basic emotions). In the future, emotional evaluations/reactions as well as psychophysiological responses to different threatening scenarios should be empirically assessed. A second nuanced difference concerns the timescale considered in our model and appraisal theories: an “end point” is reached in our model when a first behavioral response is made. This occurs relatively early, especially compared to the complex psychological processes most appraisal theories describe. It is important to keep in mind that all of our data relies solely on descriptions of threat, and on verbal report of what people would do, which may further simplify and truncate the decision process.

#### Individual differences and psychopathology

Individual differences extending to impairment offer insight into the relationship between nodes. Each node in the tree will be given different weights, according to individual differences, including trait and personality differences [22] and personal experience [56], including prior exposure to or knowledge about “appropriate” responses to a specific scenario, for example, the “correct” response to a hurricane. However, a node can also be broken. Psychiatric illnesses may be “linked to aberrant processing of environmental uncertainty” [57] and amygdala lesions in rhesus macaques [58] affect contextual modulation to certain social threat cues, like eye gaze, while amygdala lesions influence approach behaviors in humans [59]. In healthy adults, there is individual variation in peripersonal space around the face according to variations in trait anxiety scores [60], suggesting that individuals will respond variably to cues like distance in our decision tree model.

Anxiety disorders are of special interest for this model. It is known that individuals with anxiety attend to threats differently: a metanalysis [61] showed that threat-related attentional bias is a robust phenomenon across many types of anxious individuals but not in non-anxious individuals. The type of threat that is overly attended seems to be affected by type of anxiety: individuals with panic disorder are sensitive to physical threats [62] while those with social anxiety are selectively sensitive to social threats [63, 64]. There is also individual variation in sensitivity to threat in other primates: a cognitive bias to social threat develops between 3 and 9 months of age in rhesus macaques, and is sensitive to the social rank of and protectiveness of their mothers, with infants of high status and more protective mothers being more vigilant towards social threats [65]. On the other hand, oxytocin, a neuropeptide known to mediate pro-social behaviors, decreases social vigilance in adult male macaques [66]. Male and female rhesus macaques show differential response profiles to social threat, including greater high-risk aggression and gregariousness/boldness in males than females [67], mimicking gender differences observed in our decision three. In macaques, this gender effect interacted with the expression of the serotonin transporter-linked polymorphism (5-HTTLPR) and early environmental exposure to adversity in development [67]. Together, these findings support future investigation of individual differences in threat assessment, including gender, environmental, personality, and other individual differences, which we hope to ultimate relate to neural and genotype differences.

#### Moving forward

Finally, it is worth returning to Darwin’s belief that emotional behaviors could be classified across species. Although there are of course well known problems in anthropomorphizing the subjective feelings of emotions in nonhuman animals, characterizing the structure of context-dependent stimuli and emotional behaviors across species is a high priority in animal models of psychiatric illness, and methods for the behavioral phenotyping of rodents [68, 69] are well established. There are now a wealth of genetic and optogenetic manipulations in rodents that all inform mood and anxiety disorders in humans. To utilize the data from these animal models, it is essential to be able to map particular types of emotional behaviors from rodents to humans [70, 71], which may well require a shift towards focusing on both physiological and behavioral changes across species [72]. We would hope that characterizations such as the decision tree derived in the present study (Fig 3) could be developed for such comparisons, linking components of emotional behaviors and their possible pathology across species.

## Supporting Information

S1 Fig Distribution of Factor Scores. Histograms show the number of scenarios that received an average factor ratings corresponding to a score of 1(low) to 5 (high) for all 10 factors.
(EPS)

S1 Table Threat Scenarios Presented to Subjects. Each scenario is assigned a brief descriptor and label, used throughout the paper. N = Natural; A = Animal; P = Physical; S = Psychological. All Physical scenarios taken from [2].
(DOCX)
