# Supplementary material for: Exploring the Structure of Human Defensive Responses from Judgments of Threat Scenarios
Source: PLoS One. 2015 Aug 21;10(8):e0133682. doi: 10.1371/journal.pone.0133682 (PMC4546605; doi:10.1371/journal.pone.0133682)
Supplement: S1 Table — Each scenario is assigned a brief descriptor and label, used throughout the paper. N = Natural; A = Animal; P = Physical; S = Psychological. All Physical scenarios taken from [2]. (DOCX) [file pone.0133682.s002.docx]

S1 Table**.** **Threat Scenarios Presented to Subjects.** Each scenario is assigned a brief descriptor and label, used throughout the paper. N = Natural; A = Animal; P = Physical; S = Psychological. All Physical scenarios taken from [[2](#_ENREF_2)].

| Descriptor | Scenario | Label |
| --- | --- | --- |
| Hurricane  10 min | Imagine you are living in New York City, and you hear on the news that a new hurricane is arriving in 10 minutes. It is going to hit the city any moment now. This one is going to be even bigger than Hurricane Sandy, and no one knows what to make of it. | N1 |
| Hurricane  24 hr | Imagine you are living in New York City, and you hear on the news that a new hurricane is coming tonight. This one is going to be even bigger than Hurricane Sandy, and no one knows what to make of it. | N2 |
| Tornado  10 min | Imagine you are living in Kansas, and you hear on the news that a tornado is approaching your town in the next 10 minutes. You couldn't have anticipated this, and this is an emergency. You live on the countryside, and it takes at least 1 hour to reach another house or any form of help. | N3 |
| Tornado  24 hr | Imagine you are living in Kansas, and you hear on the news that a tornado is approaching your town tonight. You live on the countryside, and it takes at least 1 hour to reach another house or any form of help. | N4 |
| Bear  50 yds | You are camping in the mountains. You go out by yourself to take a walk, and you suddenly see a bear approaching from 50 yards away. | A1 |
| Bear  1 yd | You are camping in the mountains. You go out by yourself to take a walk, and you suddenly see a bear approaching from 1 yard away. | A2 |
| Bear  10 yds | You are camping in the mountains. You go out by yourself to take a walk, and you suddenly see a bear approaching from 10 yards away. | A3 |
| Shark  100 yds | You are swimming near the beach, and you suddenly realize there is some animal that is approaching you from 100 yards away.  It may be a shark but you are unsure. | A4 |
| Shark  10 yds | You are swimming near the beach, and you suddenly realize there is some animal that is approaching you from 10 yards away.  It may be a shark but you are unsure. | A5 |
| Whisper | Alone at home one night, you have settled down to read a book when you hear some movement right outside of your window.  You cannot see anything, but when you listen more closely, it sounds like people whispering. | P1 |
|  |  |  |
| Elevator | You are alone in an elevator late at night.  As it stops and the doors open, a menacing stranger rushes in to attack you, blocking the door. | P2 |
|  |  |  |
| Stoplight | You are alone in a car on your way home.  While stopped at a traffic signal, an angry stranger begins banging on your car window and yelling threatening things at you. | P3 |
| Tailgating | Driving along a two-lane road, you see in your rear-view mirror that a car is dangerously tailgating you.  They cannot pass and begin honking their horn aggressively at you while continuing to follow too closely. | P4 |
| Corner | It is past midnight and you are walking through an unfamiliar part of town.  As you round a corner, you accidentally run into a man.  He becomes angry and shoves you. | P5 |
| Acquaintance | You and someone you do not really know that well are standing around and talking in an empty parking lot.  The acquaintance begins to shove and push you.  You are unsure whether s/he (same sex as you,) is serious or just kidding around. | P6 |
| Park | You are outside in a park area at night when you see a menacing stranger with a knife about 30 feet away directly approaching you.  It is obvious the person is planning to attack you. | P7 |
|  |  |  |
| Grab | You are alone as you exit an empty campus building late one night.  Just as you get outside you feel a hand grab your arm. | P8 |
|  |  |  |
| Noise | You are sleeping in bed during the night, but suddenly wake up thinking you have heard a suspicious noise.  It is dark and you are alone. | P9 |
|  |  |  |
| Phone | You are alone at home one night about to go to bed when the phone rings.  You answer it, and there is an unfamiliar voice on the other end.  It tells you that he or she is right outside of your house and hangs up. | P10 |
| Bomb | Coming home one day, you find an unexpected shoe-box-sized package waiting for you by the mailbox.  As you sit down to open it, you notice a faint ticking sound that appears to come from inside the package. | P11 |
|  |  |  |
| Blackmail  Email | One of your colleagues has blackmailed you via e-mail, saying that he or she will spread rumors about you.  The rumor is an embarrassing one that deals with your personal issues. | S1 |
|  |  |  |
| Blackmail  Face | You are taking a break at work and one of your co-workers approaches you.  He/she is blackmailing you face-to-face.  He or she threatens to spread rumors about your personal issues. | S2 |
| Boss | It is almost the holiday season and you are planning to take a few days off from work.  You bring this up at your weekly meeting, and your boss disapproves.  Your immediate boss threatens to fire you if you take a break. | S3 |
|  |  |  |
| Rumor | Recently, you have noticed that one of your co-workers have been talking behind your back at work.  He/she has been spreading rumors, and seems to drop negative remarks about you to your immediate boss as well. | S4 |
| Cellphone | You are working for a cellphone company, and you have had a new design for a phone in mind.  You decide to share it with a colleague.  You tell him/her that this may be revolutionary and that he/she should keep it confidential.  Sooner or later, you realize that the colleague has already spread the idea and has taken credit for it. | S5 |
| Party | Your close friend is having a holiday party, and has not invited you to this party. | S6 |
| Bar | A group of your colleagues go out to a bar after work, but no one asks you to join them. | S7 |
| Homophobic | Imagine you are secretly gay and a close family member is homophobic.  During a family party, they make a derogatory comment toward gay people. | S8 |
| Political | You realize that you and your boss hold very different political views.  After work, you and your boss get together and he/she asks about your political views. | S9 |
